# Supplementary material for: Identification and Characterization of Aspergillus nidulans Mutants Impaired in Asexual Development under Phosphate Stress
Source: Cells. 2019 Nov 26;8(12):1520. doi: 10.3390/cells8121520 (PMC6952808; doi:10.3390/cells8121520)
Supplement: Supplementary file 1 [file cells-08-01520-s001.zip › Supplementary_Files/Supplementary File 1_2019_09_27.pdf]

Exonic mutations found in FLIP166 by comparison of DNAseq reads with the reference used in the IGV software (FGSCA4), on the one hand, and RNA-seq reads of reference and Δ*flbB* strains, or reference and Δ*sltA* strains (EA. Espeso; unpublished), on the other hand:

1) An6835 (ChrI): Cytochrome P450 (CYP505A8)

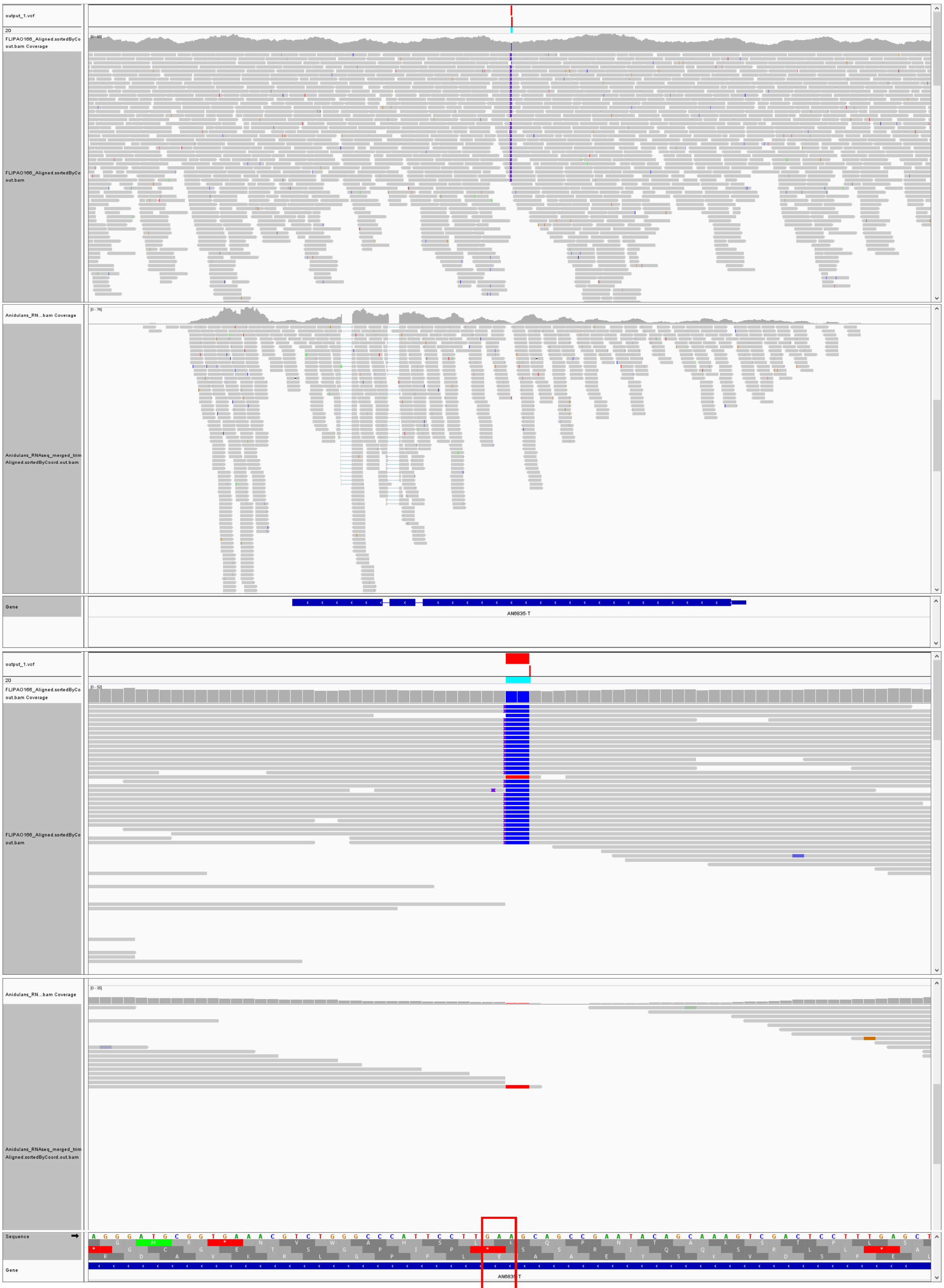

Modification in codon 588. TTC (Phe) in FGSCA4, AAC (Asn) in RNA-seq reads, GGC-AAC (Gly-Asn) in FLIP166. Candidate mutation discarded by Sanger sequencing.

2) An12172 (ChrII): LCCL domain-containing TM protein.

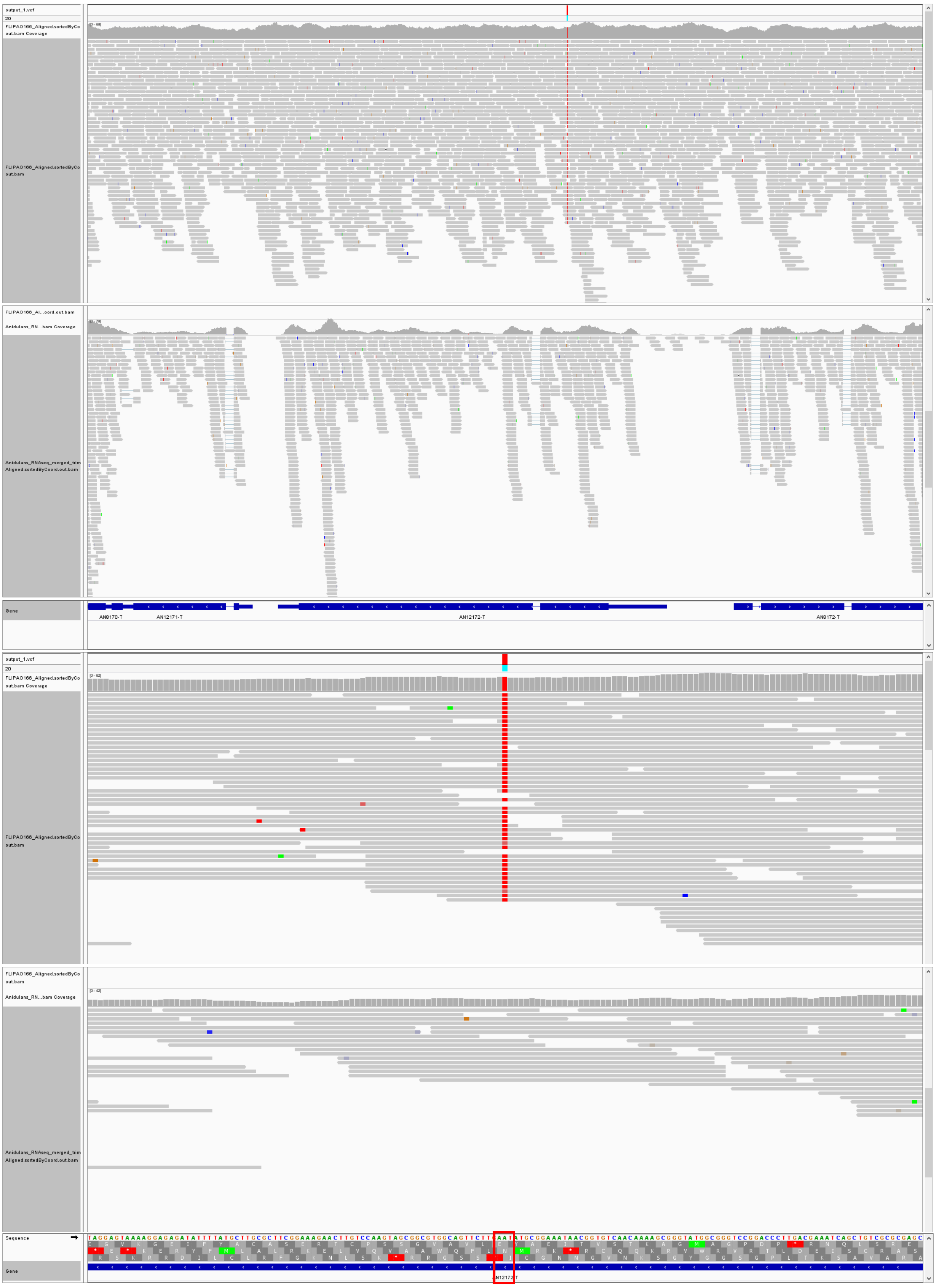

Point modification in codon 94. ATT (Ile) in FGSCA4 and RNA-seq reads, AAT (Asn) in FLIP166. Mutation confirmed by Sanger sequencing.

3) An8821 (ChrIII): Uncharacterized.

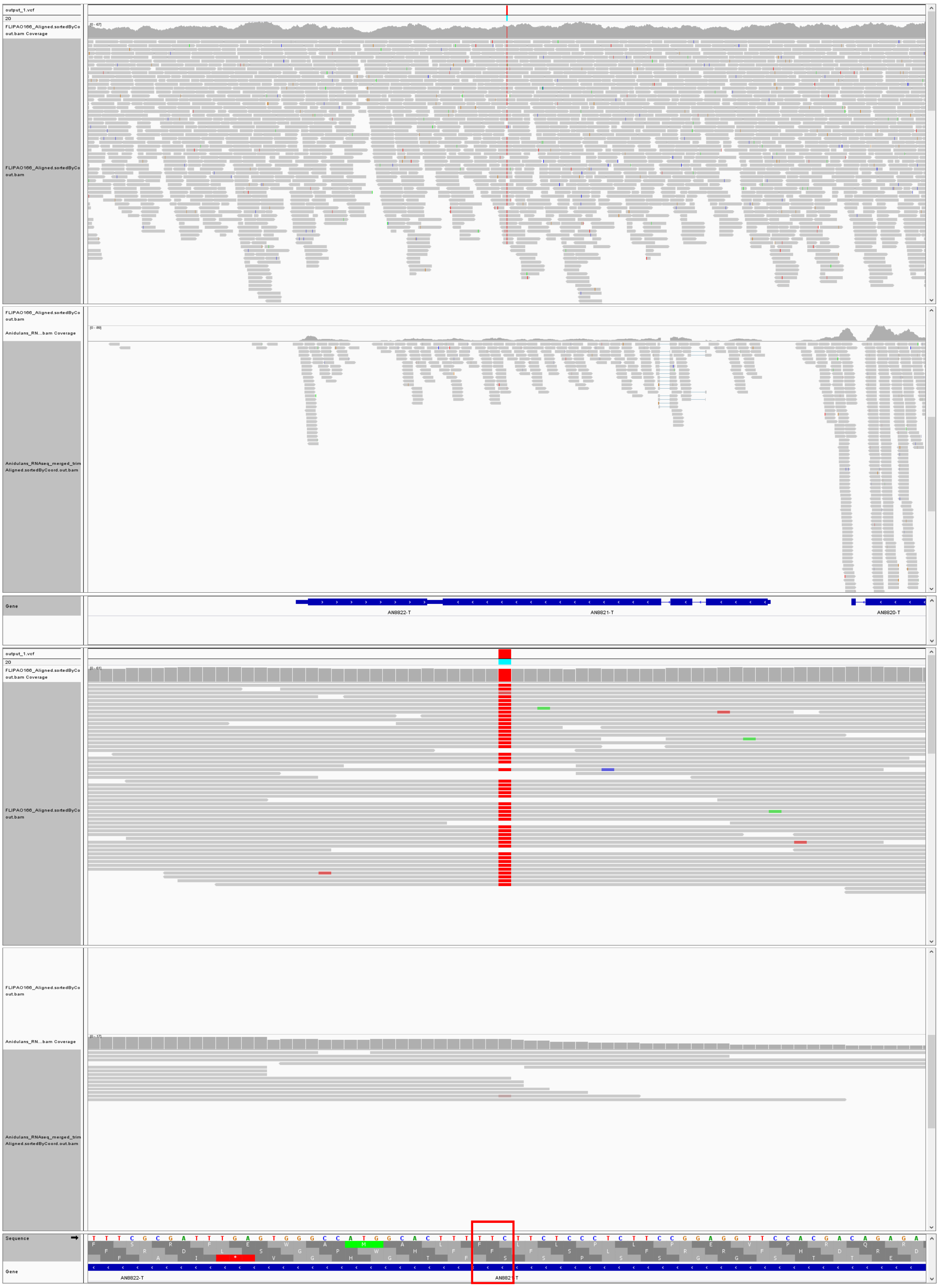

Point modification in codon 515. GAA (Glu) in FGSCA4 and RNA-seq reads, to AAA (Lys) in FLIP166. Mutation confirmed by Sanger sequencing.

4) An8615 (ChrIII): Cytochrome P450 (CYP677A1)

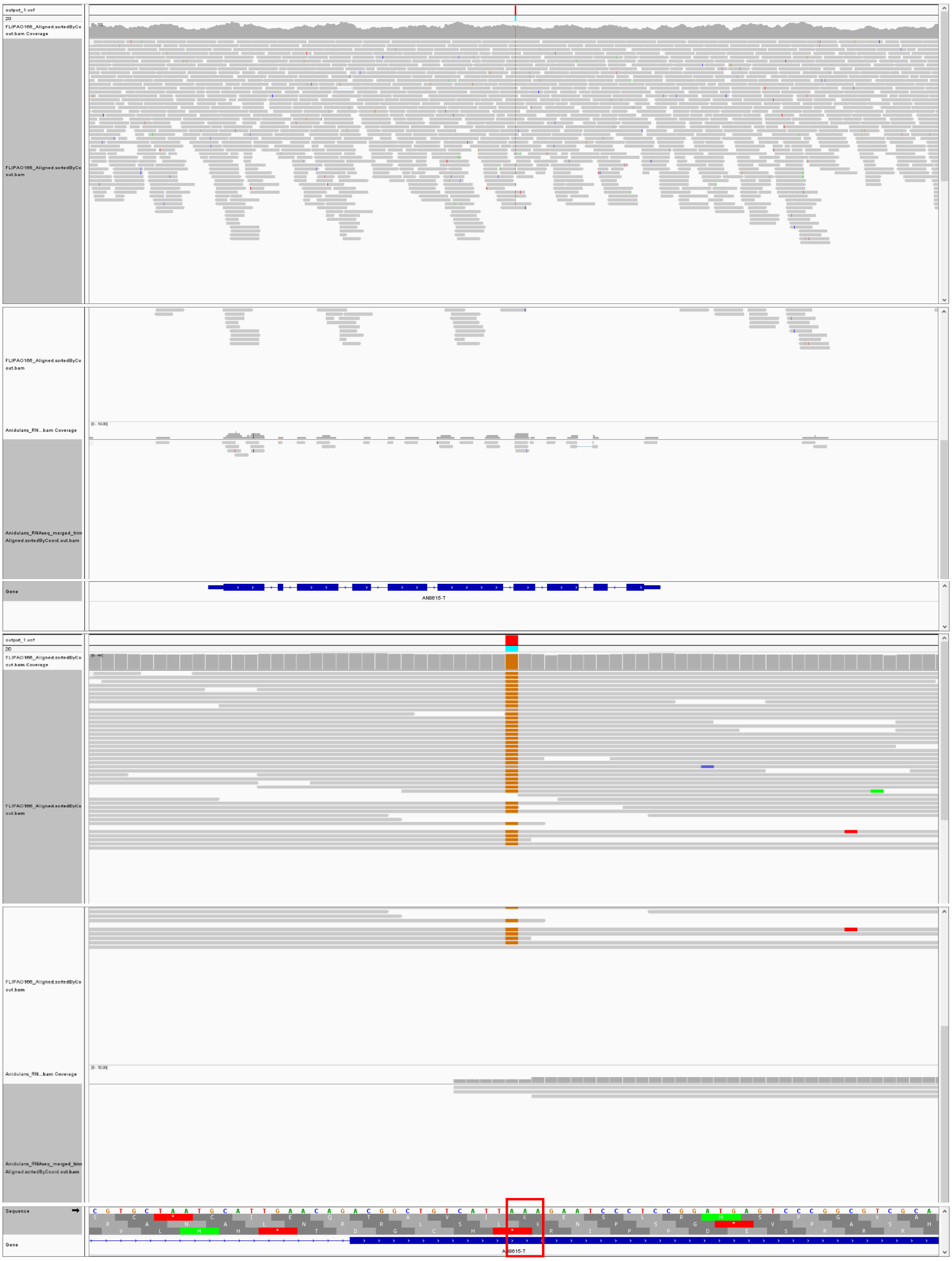

Point modification in codon 363. AAA (Lys) in FGSCA4 and RNA-seq reads, to GAA (Glu) in FLIP166. Mutation confirmed by Sanger sequencing.

5) An7222 (ChrIV): NACHT domain-containing protein.

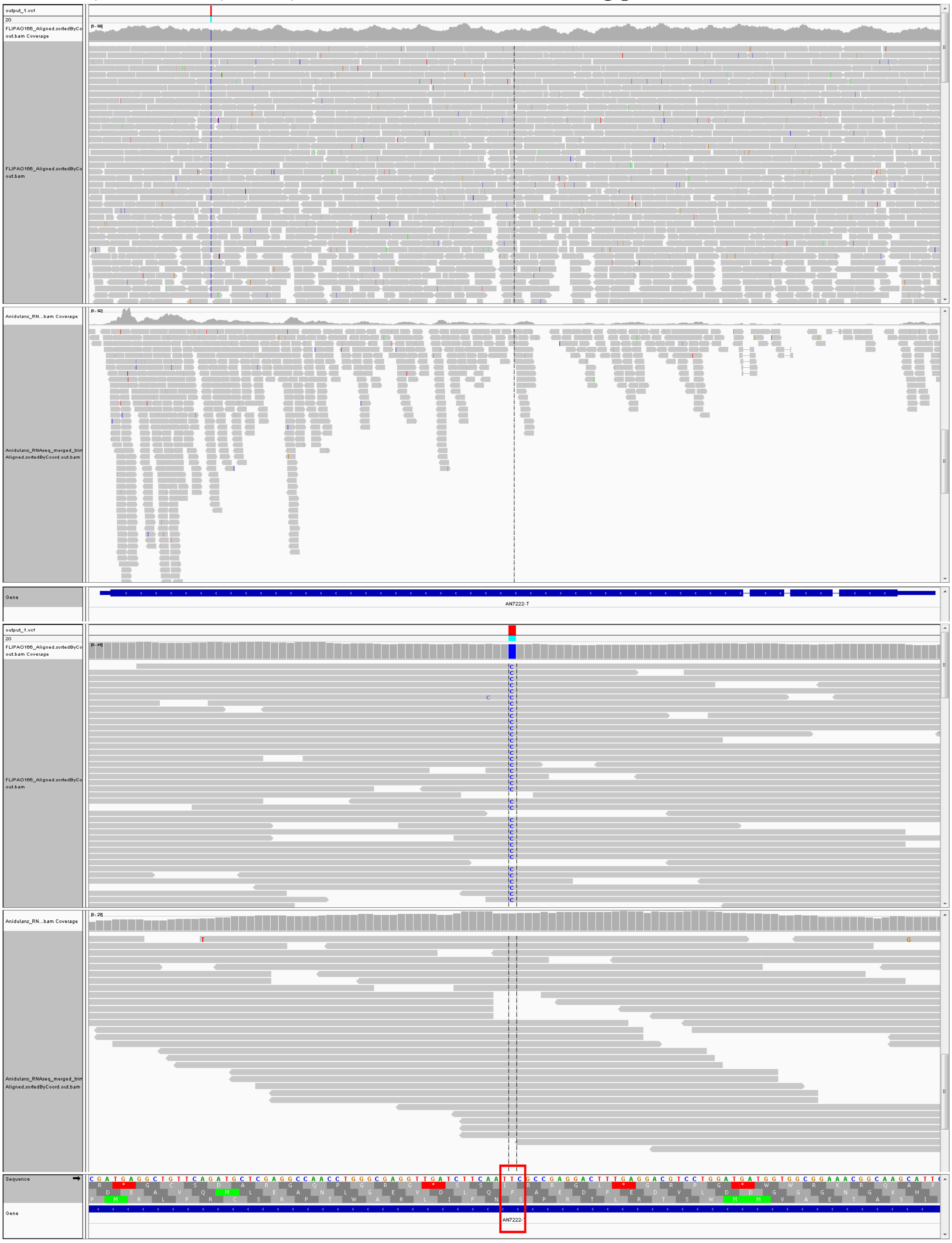

Point modification in codon 1793. GAA (Glu) in FGSCA4 and RNA-seq reads, to GGA (Gly) in FLIP166. Mutation confirmed by Sanger sequencing.

6) An1459 (ChrVII): Protein O-mannosyltranferase (PmtC), involved in hyphal growth and conidia formation. Transmembrane protein.

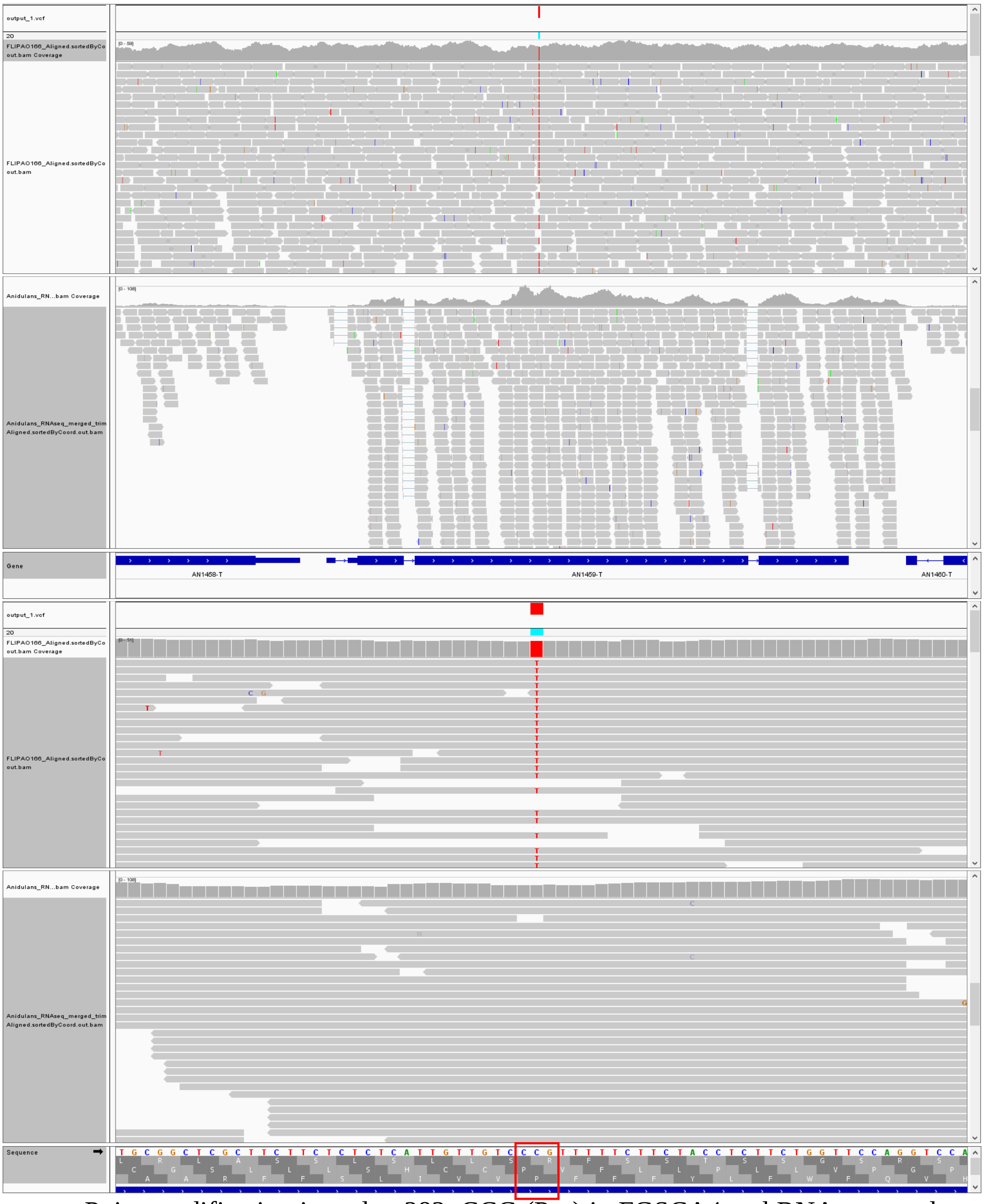

Point modification in codon 282. CCG (Pro) in FGSCA4 and RNA-seq reads, to CTG (Leu) in FLIP166. Mutation confirmed by Sanger sequencing.

7) An12335 (ChrVII): Acyl-CoA dehydrogenase (AcdA)

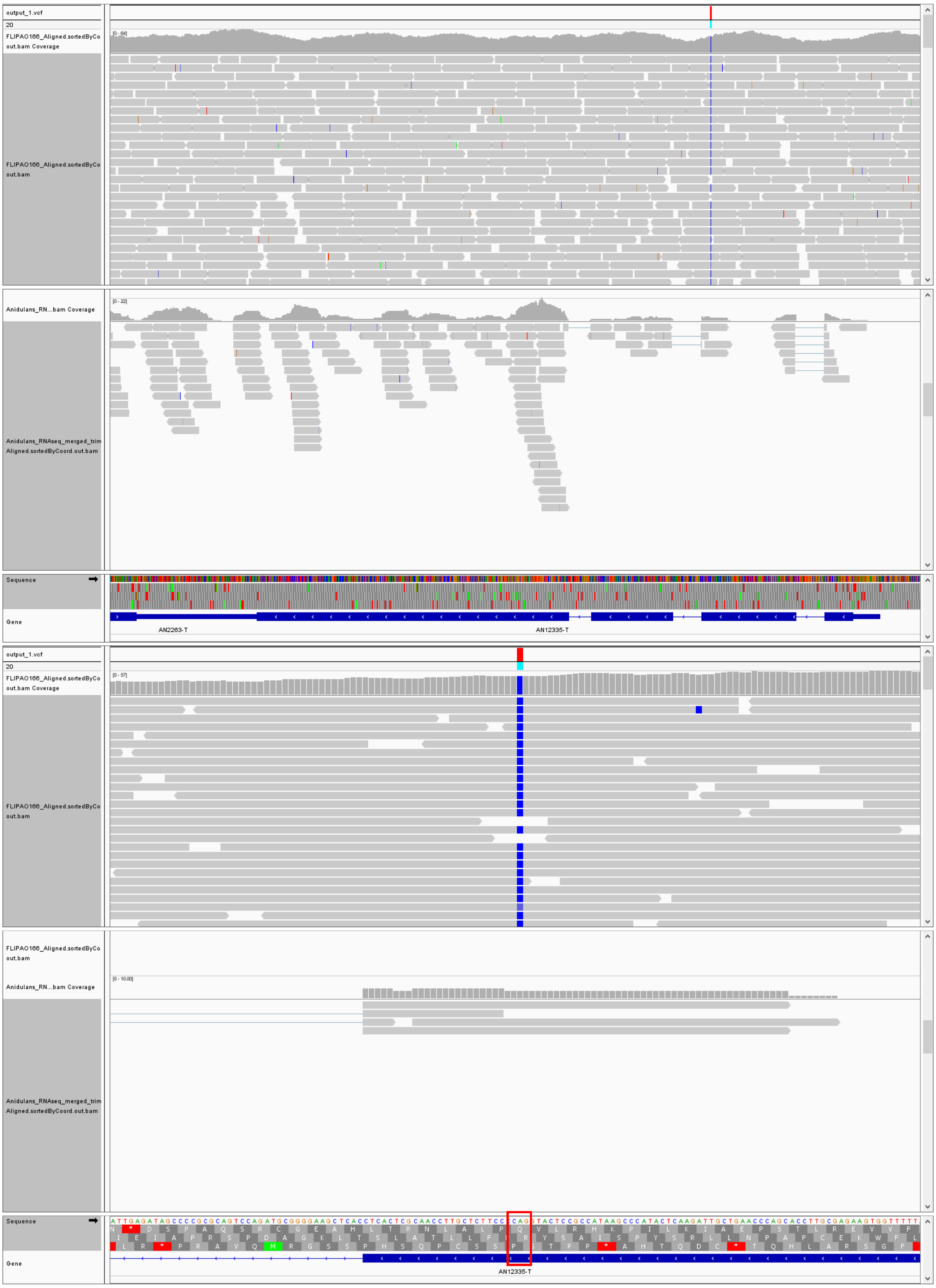

Point modification in codon 99. CTG (Leu) in FGSCA4 and RNA-seq reads, to CGG (Arg) in FLIP166. Mutation not confirmed by Sanger sequencing.

8) An1305 (ChrVIII); Domain with nucleotidyltransferase activity.

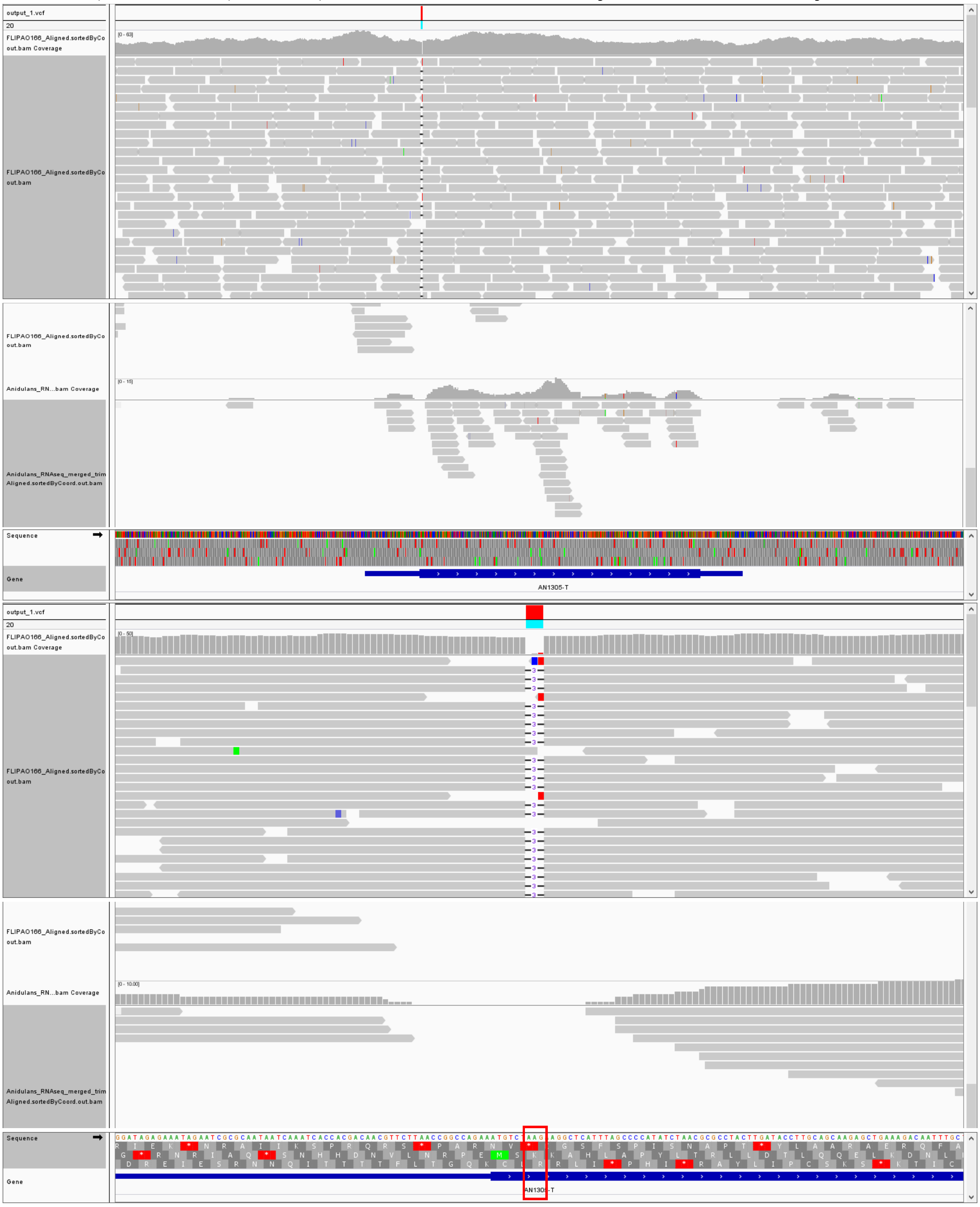

No RNAseq reads in this region. Possible deletion of codon 3 (AAG; Lys) without altering the reading frame. Mutation not confirmed by Sanger sequencing.

9) An0637 (ChrVIII): Helicase.

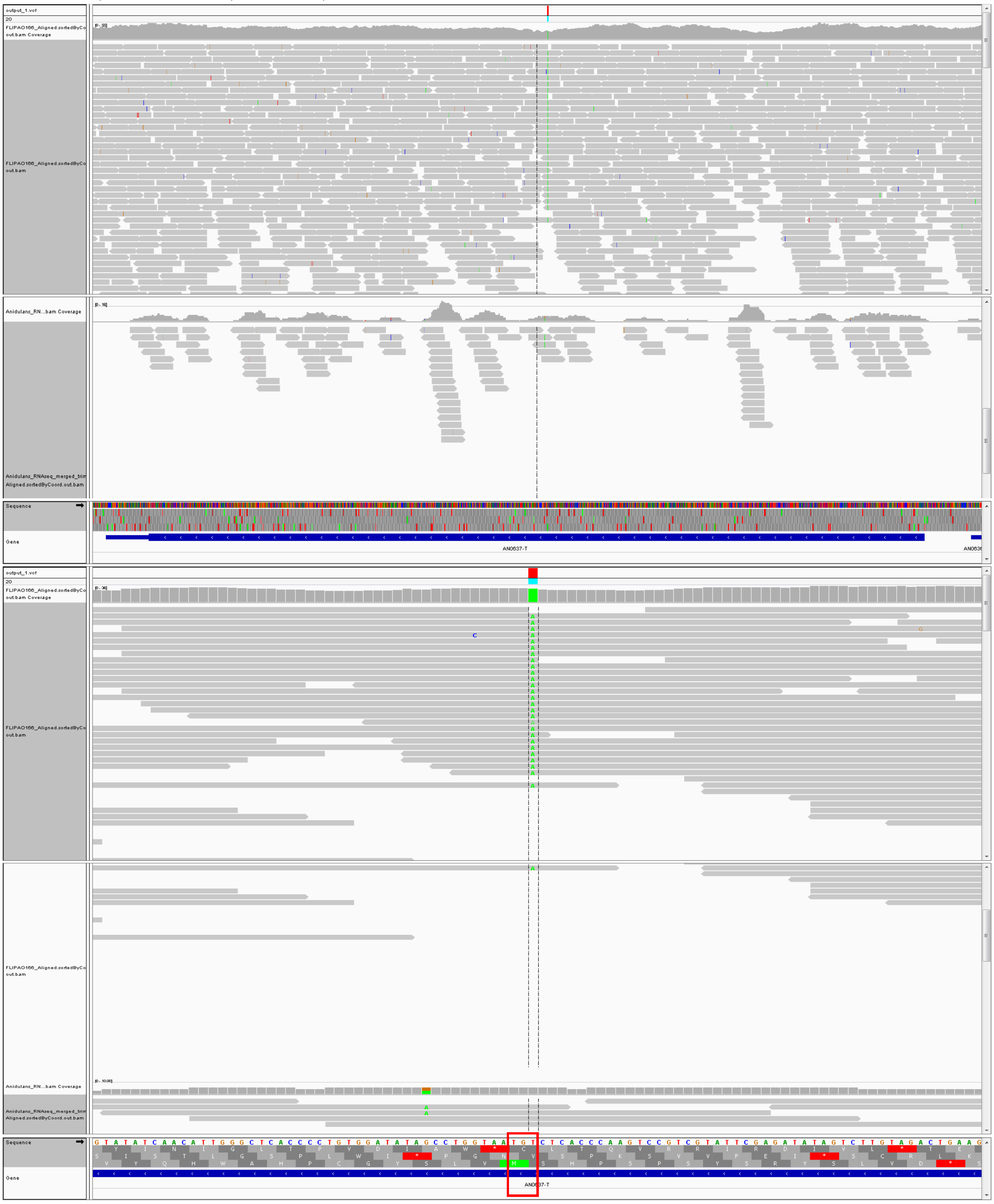

Point modification in codon 415. ACA (Thr) in FGSCA4 and RNA-seq reads, to TCA (Ser) in FLIP166. Mutation not confirmed by Sanger sequencing.

10) An0560 (ChrVIII): Ortholog(s) have role in Golgi to plasma membrane transport, exocyst assembly, exocyst localization, spliceosomal complex assembly.

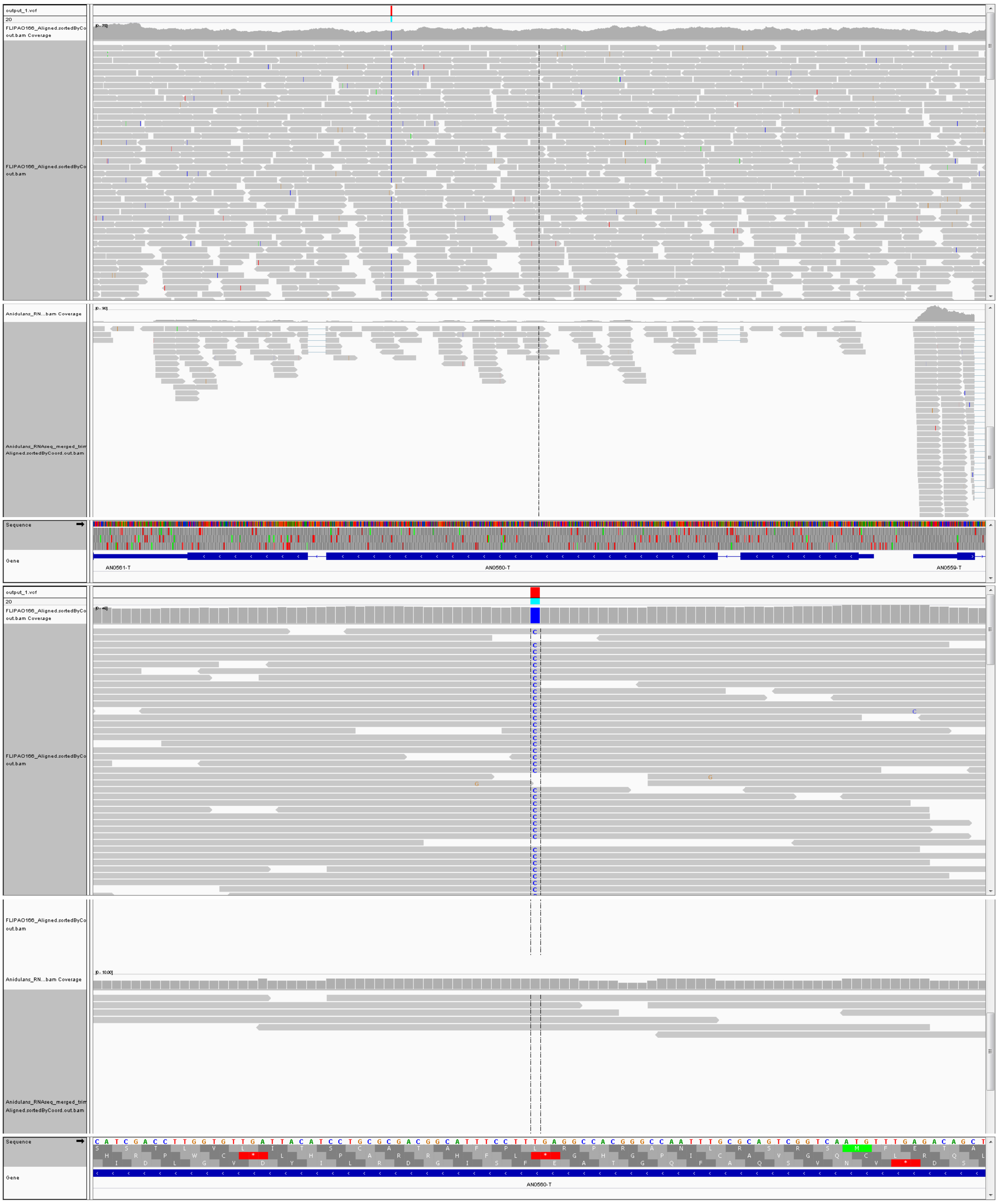

Point modification in codon 488. AAA (Lys) in FGSCA4 and RNA-seq reads, to GAA (Glu) in FLIP166. Mutation confirmed by Sanger sequencing.
