# Supplementary material for: Identification and Characterization of Aspergillus nidulans Mutants Impaired in Asexual Development under Phosphate Stress
Source: Cells. 2019 Nov 26;8(12):1520. doi: 10.3390/cells8121520 (PMC6952808; doi:10.3390/cells8121520)
Supplement: Supplementary file 1 [file cells-08-01520-s001.zip › Supplementary_Files/Table S1_Strains.pdf]

BD177	*pyrG89; argB2; ΔflbB::riboB*^{Afum}, *pabaB22, pyroA4, ΔnkuA::argB; riboB2, veA1*	[1]
BD178	*pyrG89; argB2; pabaB22, pyroA4, ΔnkuA::argB; riboB2, ΔflbE::riboB*^{Afum}, *veA1.*	[1]
BD198	*pyrG8; argB2; pabaB22, pyroA4, ΔnkuA::argB; riboB2, ΔflbD::riboB*^{Afum},*veA1*	[2]
BD228	*pyrG89; argB2; pyroA4, ΔnkuA::argB; ΔflbC::pyrG*^{Afum}; *veA1*	[3]
BD246	*pyrG89; argB2; pyroA4, ΔnkuA::argB; flbD::gfp::pyrG*^{Afum}, *veA1*	[2]
BD274	*pyrG89; argB2; ΔflbB::riboB*^{Afum}, *pabaB22, pyroA4, ΔnkuA::argB; riboB2, veA1, ΔflbD::pyrG*^{Afum}	[2]
BD376	*pyrG89; argB2; pyroA4, ΔnkuA::argB; ΔbrlA::pyrG*^{Afum}; *veA1*	A. Garzia, unpublished
BD689	*pyrG89; ΔfluG::pyrG*^{Afum} *,argB2; pyroA4, ΔnkuA::argB; veA1*	[4]
BD950 (FLIP166)	*pyrG89; An12172*^{Ile94Asn}; *argB2; ΔflbB::riboB*^{Afum}, *pabaB22, pyroA4, ΔnkuA::argB; pmtC*^{Pro282Leu}, *acdA*^{Leu99Arg};*riboB2, veA1*	This study
BD951	*pyrG89; An12172*^{Ile94Asn} *;argB2; ΔflbB::riboB*^{Afum}, *pabaB22, pyroA4, ΔnkuA::argB; pmtC*^{Pro282Leu}, *acdA*^{Leu99Arg};*riboB2, veA1; pRG3-AMA-NotI::socA*	This study
BD955	*pyrG89; An12172*^{Ile94Asn} *;argB2; ΔflbB::riboB*^{Afum}, *pabaB22, pyroA4, ΔnkuA::argB; pmtC*^{Pro282Leu}, *acdA*^{Leu99Arg};*riboB2, veA1; pRG3-AMA-NotI::flbB*	This study
BD1172	*pyrG89; argB2; pyroA4, ΔnkuA::argB; ΔsocA::pyrG*^{Afum}; *veA1*	This study
BD1177	*pyrG89; argB2; pyroA4, ΔnkuA::argB; socA::gfp::pyrG*^{Afum}; *veA1*	This study
BD1181	*pyrG89; argB2; pyroA4, ΔnkuA::argB; gpdA*^{p}::*socA::gfp::pyrG*^{Afum}; *veA1*	This study
BD1174	*pyrG89; argB2; ΔflbB::riboB*^{Afum}, *pabaB22, pyroA4, ΔnkuA::argB; ΔsocA::pyrG*^{Afum}; *riboB2, veA1*	This study
BD1179	*pyrG89; argB2; ΔflbB::riboB*^{Afum}, *pabaB22, pyroA4, ΔnkuA::argB; socA::gfp::pyrG*^{Afum}; *riboB2, veA1*	This study
BD1183	*pyrG89; argB2; ΔflbB::riboB*^{Afum}, *pabaB22, pyroA4, ΔnkuA::argB; gpdA*^{p}::*socA::gfp::pyrG*^{Afum}; *riboB2, veA1*	This study
BD1245	*pyrG89; argB2; pyroA4, ΔnkuA::argB; socA::ha*$_{3x}$::*pyrG*^{Afum}; *veA1*	This study
BD1249	*pyrG89; argB2; ΔflbB::riboB*^{Afum}, *pabaB22, pyroA4, ΔnkuA::argB; socA::ha*$_{3x}$::*pyrG*^{Afum}; *riboB2, veA1*	This study
BD1247	*pyrG89; argB2; pyroA4, ΔnkuA::argB; gpdA*^{p}::*socA::ha*$_{3x}$::*pyrG*^{Afum}; *veA1*	This study
BD1251	*pyrG89; argB2; ΔflbB::riboB*^{Afum}, *pabaB22, pyroA4, ΔnkuA::argB; gpdA*^{p}::*socA::ha*$_{3x}$::*pyrG*^{Afum}; *riboB2, veA1*	This study
BD1291	*pyrG89; An12172::gfp::pyrG*^{Afum}; *argB2; ΔflbB::riboB*^{Afum}, *pabaB22, pyroA4, ΔnkuA::argB; pmtC*^{Pro282Leu}, *acdA*^{Leu99Arg};*riboB2, veA1*	This study
BD1294	*pyrG89; An12172::gfp::pyrG*^{Afum}; *argB2; pyroA4, ΔnkuA::argB; veA1*	This study
BD1296	*pyrG89; An12172*^{Ile94Asn}::*gfp::pyrG*^{Afum}; *argB2; pyroA4, ΔnkuA::argB; veA1*	This study

BD1299	*pyrG89; An12172::gfp::pyrG^{Afum}; argB2; ΔflbB::riboB^{Afum}, pabaB22, pyroA4, ΔnkuA::argB; riboB2, veA1*	This study
BD1302	*pyrG89; An12172^{Ile94Asn}::gfp::pyrG^{Afum}; argB2; ΔflbB::riboB^{Afum}, pabaB22, pyroA4, ΔnkuA::argB; riboB2, veA1*	This study
BD1305	*pyrG89; An12172^{Ile94Asn}; argB2; ΔflbB::riboB^{Afum}, pabaB22, pyroA4, ΔnkuA::argB; pmtC^{Pro282Leu}, acdA::gfp::pyrG^{Afum}; riboB2, veA1*	This study
BD1308	*pyrG89; argB2; pyroA4, ΔnkuA::argB; acdA::gfp::pyrG^{Afum}; veA1*	This study
BD1310	*pyrG89; argB2; pyroA4, ΔnkuA::argB; acdA^{Leu99Arg}::gfp::pyrG^{Afum}; veA1*	This study
BD1313	*pyrG89; argB2; ΔflbB::riboB^{Afum}, pabaB22, pyroA4, ΔnkuA::argB; acdA::gfp::pyrG^{Afum}; riboB2, veA1*	This study
BD1316	*pyrG89; argB2; ΔflbB::riboB^{Afum}, pabaB22, pyroA4, ΔnkuA::argB; acdA^{Leu99Arg}::gfp::pyrG^{Afum}; riboB2, veA1*	This study
BD1319	*pyrG89; An12172^{Ile94Asn} ;argB2; ΔflbB::riboB^{Afum}, pabaB22, pyroA4, ΔnkuA::argB; pmtC::gfp::pyrG^{Afum}, acdA^{Leu99Arg}; riboB2, veA1*	This study
BD1323	*pyrG89; argB2; pyroA4, ΔnkuA::argB; pmtC::gfp::pyrG^{Afum}; veA1*	This study
BD1327	*pyrG89; argB2; pyroA4, ΔnkuA::argB; pmtC^{Pro282Leu}::gfp::pyrG^{Afum}; veA1*	This study
BD1330	*pyrG89; argB2; ΔflbB::riboB^{Afum}, pabaB22, pyroA4, ΔnkuA::argB; pmtC::gfp::pyrG^{Afum}; riboB2, veA1*	This study
BD1333	*pyrG89; argB2; ΔflbB::riboB^{Afum}, pabaB22, pyroA4, ΔnkuA::argB; pmtC^{Pro282Leu}::gfp::pyrG^{Afum}; riboB2, veA1*	This study
BD1351	*pyrG89; argB2; ΔflbB::riboB^{Afum}, pabaB22, pyroA4, ΔnkuA::argB; pmtC^{Arg762Gly}::gfp::pyrG^{Afum}; riboB2, veA1*	This study
BD1352	*pyrG89; argB2; ΔflbB::riboB^{Afum}, pabaB22, pyroA4, ΔnkuA::argB; pmtC^{Tyr767Stop}::gfp::pyrG^{Afum}; riboB2, veA1*	This study
CΔTMPBP1	*pabaA1, yA2; ΔtmpB::riboB*	[5]
FGSC26	*biA1 veA1*	[6]
FGSCA68	*suA1adE20, yA2, adE20; acrA1; phenA2; pyroA4; lysB5; sB3; nicB8; riboB2, veA1*	[6]
FGSCA283	*suA1adE20 yA2 adE20 acrA1 galA1 pyroA4 (ssb+) facA303 sB3 nicB8 riboB2 veA1*	Fungal Gen. Stock Center
FLIP mutants	*pyrG89; argB2; ΔflbB::riboB^{Afum}, pabaB22, pyroA4, ΔnkuA::argB;riboB2, veA1; unknown mutation(s)*	This study
RMSO11	*pabaA1 yA2 ΔargB::trpCΔB trpC801 veA1*	[7]
TGS6	*pabaA1, yA2; ΔargB::trpCΔB; trpC801, ΔtmpA::argB, veA1*	[8]
TN02A3	*pyrG89; argB2; pyroA4, ΔnkuA::argB; veA1*	[9]

Table S2: Strains of *Aspergillus nidulans* used in this study.

1. Garzia, A.; Etxebeste, O.; Herrero-Garcia, E.; Fischer, R.; Espeso, E. A.; Ugalde, U. *Aspergillus nidulans* FlbE is an upstream developmental activator of conidiation functionally associated with the putative transcription factor FlbB. *Mol. Microbiol.* **2009**, *71*, 172–184.

2. Garzia, A.; Etxebeste, O.; Herrero-García, E.; Ugalde, U.; Espeso, E. A. The concerted action of bZip and cMyb transcription factors FlbB and FlbD induces *brlA* expression and asexual development in *Aspergillus nidulans*. *Mol. Microbiol.* **2010**, *75*, 1314–1324.
3. Kwon, N.-J.; Garzia, A.; Espeso, E. A.; Ugalde, U.; Yu, J.-H. FlbC is a putative nuclear C2H2 transcription factor regulating development in *Aspergillus nidulans*. *Mol. Microbiol.* **2010**, *77*, 1203–1219, doi:10.1111/j.1365-2958.2010.07282.x.
4. Iradi-Serrano, M.; Tola-García, L.; Cortese, M. S.; Ugalde, U. The early asexual development regulator *fluG* codes for a putative bifunctional enzyme. *Front. Microbiol.* **2019**, *10*, 778, doi:10.3389/fmicb.2019.00778.
5. Soid-Raggi, G.; Sánchez, O.; Ramos-Balderas, J. L.; Aguirre, J. The Adenylate-Forming Enzymes AfeA and TmpB Are Involved in *Aspergillus nidulans* Self-Communication during Asexual Development. *Front. Microbiol.* **2016**, *7*, 353, doi:10.3389/fmicb.2016.00353.
6. Käfer, E. Origins of translocations in *Aspergillus nidulans*. *Genetics* **1965**, *52*, 217 LP-232.
7. Stringer, M. A.; Dean, R. A.; Sewall, T. C.; Timberlake, W. E. Rodletless, a new *Aspergillus* developmental mutant induced by directed gene inactivation. *Genes Dev.* **1991**, *5*, 1161–1171, doi:10.1101/gad.5.7.1161.
8. Soid-Raggi, G.; Sánchez, O.; Aguirre, J. TmpA, a member of a novel family of putative membrane flavoproteins, regulates asexual development in *Aspergillus nidulans*. *Mol. Microbiol.* **2005**, *59*, 854–869, doi:10.1111/j.1365-2958.2005.04996.x.
9. Nayak, T.; Szewczyk, E.; Oakley, C. E.; Osmani, A.; Ukil, L.; Murray, S. L.; Hynes, M. J.; Osmani, S. A.; Oakley, B. R. A versatile and efficient gene-targeting system for *Aspergillus nidulans*. *Genetics* **2006**, *172*, 1557–66.
