# Supplementary material for: Identification and Characterization of Aspergillus nidulans Mutants Impaired in Asexual Development under Phosphate Stress
Source: Cells. 2019 Nov 26;8(12):1520. doi: 10.3390/cells8121520 (PMC6952808; doi:10.3390/cells8121520)
Supplement: Supplementary file 1 [file cells-08-01520-s001.zip › Supplementary_Files/Table S2_Oligonucleotides.pdf]

Supplementary Table 2: Oligonucleotides used in this study.

Name	Sequence (5’→3’)	Objective
flbD-PP1	CGACTCCATGACCACATTCC	Amplification of *flbD::gfp::pyrG*^{Afum} construct
flbD-GSP4	GGCAGGCGTTACTGTATACGT	
pRG3Up	GAATTCGAGCTCGGTACC	Sequencing of the flanking regions of the insert of pRG3
pRG3Down	AAGCTTGCATGCGCGGCC	
An8501-sek1	CGTTGGTCCGTTTCGCATGAAGC	Sequencing of the ORF of *An8501/socA*
An8501-sek2	CGTGTTATCGCCGAGATCAGAGG	
An8501-sek3	CGTCACTCATACACGTAGCCACCC	
An8501-sek4	GCGAAGCGATCTGTGAGGTCC	
An8501-PP1	CGACGAAGGCCCATACCATCC	Deletion and tagging of *An8501/socA* with *gfp*^{Afum} or ha_{3x} (native promoter or constitutive *gpdA*^{p} promoter)
An8501-PP2	CATGAGCAGCGCCCGTCAATGG	
An8501-GSP3	TAAGTGTTGGTGCACTTCCGACACG	
An8501-GSP4	GCAGCAGCCCATCTCGACTTCC	
An8501-GSP1	GAGACCAACAACGTCGGCGG	
An8501-GSP2	GACGCTCGTCCTAGGAGGCG	
An8501-SMP1	CCATTGACGGGCGCTGCTCATGACCGGTCGCCT CAAACAATGCTCT	
An8501-GFP1	CGCCTCCTAGGACGAGCGTCGGAGCTGGTGCAG GCGCTGGAGCC	
An8501-GFP2	CGTGTCGGAAGTGCACCAACACTTAGTCTGAGA GGAGGCACTGATGCG	
An8501-geneSP	ATGTCGGAAACTCTCCCCAGACG	
An8501-PP2´-ATG	GAGCAGCGCCCGTCAATGG	
An8501-gpdAUp	CCATTGACGGGCGCTGCTCCCATCCGGTGCTCTG CACTCGACC	
An8501-gpdADw	CGTCTGGGGAGAGTTTCCGACATGGTGATGTCT GCTCAAGCGGGG	
An6835-Up	CTTGCATTGGACGACC	Sequencing of FLIP166´s mutation in *An6835*
An6835-Dw	CAGGACAGCTTGAACTGC	
An12172-Up	CAGACGCCACCCTCC	Sequencing of FLIP166´s mutation in *An12172*
An12172-Dw	CTCCTGCATGGATAGCG	
An8615-Up	CGAAACTGTCGAAGATCC	Sequencing of FLIP166´s mutation in *An8615*
An8615-Dw	CTTGACGGATCAATCTGC	
An1459-Up	GTGGGCTATGACGGC	Sequencing of FLIP166´s mutation in *An1459/pmtC*
An1459-Dw	CGGCTAGCTCGTGCG	
An0560-Up	CCCAACTAAGCTGATTGCG	Sequencing of FLIP166´s mutation in *An0560*
An0560-dw	CCAGCCTGTTCGTGG	
An7222-Up	GCCAGACTTCCACGG	Sequencing of FLIP166´s mutation in *An7222*
An7222-Dw	GGTCGTCACTAAGGGCG	
An8821-Up	GGATGGAACAGCAGTGG	Sequencing of FLIP166´s mutation in *An8821*
An8821-Dw	GGTGTTCCAGAGGGGG	
An12172-GSP1	GTGGTTACGGGAGTCTTCCAAGTCTTTTCG	Tagging of WT or FLIP166´s-mutant
An12172-GSP2	GTTTTTCGGCTGTGGAGGAGAAATCCAGC	
AN12172-GSP3	TAGATAACCGTCTTTATTAGAAGTACTGTGC	

AN12172-GSP4	CTGAAGACCTCTGGCCCAATGC	versions of *An12172* with *gfp*^{Afum}
AN12172-GFP1	GCTGGATTTCTCCTCCACAGCCGAAAAACGGAG CTGGTGCAGGCGCTGGAGCC	
AN12172-GFP2	GCACAGTACTTCTAATAAAGACGGTTATCTAGT CTGAGAGGAGGCACTGATGCG	
An12335-GSP1	CCTCCACTCCGAACAACCAAAAGTCTAACC	Tagging of WT or FLIP166´s-mutant versions of *An12335/acdA* with *gfp*^{Afum}
An12335-GSP2	GAGAAGACTCTTCAACTTCGAGAACCCATTCGC	
AN12335-GSP3	TAGCTTTGGCTCCTTCGATAGCGTTGTGACG	
AN12335-GSP4	GTTTCTTTTCCGCACAGATGGCTAAAGAGGC	
AN12335-GFP1	GCGAATGGGTTCTCGAAGTTGAAGAGTCTTCTC GGAGCTGGTGCAGGCGCTGGAGCC	
AN12335-GFP2	CGTCACAACGCTATCGAAGGAGCCAAAGCTAGT CTGAGAGGAGGCACTGATGCG	
PmtC-GSP1	CTGGTCATCACTATGGCCCTGAGC	Tagging of WT or FLIP166´s-mutant versions of *An1459/pmtC* with *gfp*^{Afum}
PmtC-GSP2	TTTCGCGAAGTGCAAGTCATAGCCAAGC	
PmtC-GSP3	TAGGAGCATCTGATCTCATCTCGTTCTCTCCC	
PmtC-GSP4	CGCTGTTGTAGCCGATGAAACTGC	
PmtC-GFP1	GCTTGGCTATGACTTGCACTTCGCGAAAGGAGC TGGTGCAGGCGCTGGAGCC	
PmtC-GFP2	GGGAGAGAACGAGATGAGATCAGATGCTCCTAG TCTGAGAGGAGGCACTGATGCG	
PmtC-sek1	CAGGAACACCTCGATCC	Sequencing of the ORF of *An1459/pmtC*
PmtC-sek4	CACCGGCGAACACAGC	
