# Supplementary figures and images for: Identification and Characterization of Aspergillus nidulans Mutants Impaired in Asexual Development under Phosphate Stress
Source: Cells. 2019 Nov 26;8(12):1520. doi: 10.3390/cells8121520 (PMC6952808; doi:10.3390/cells8121520)

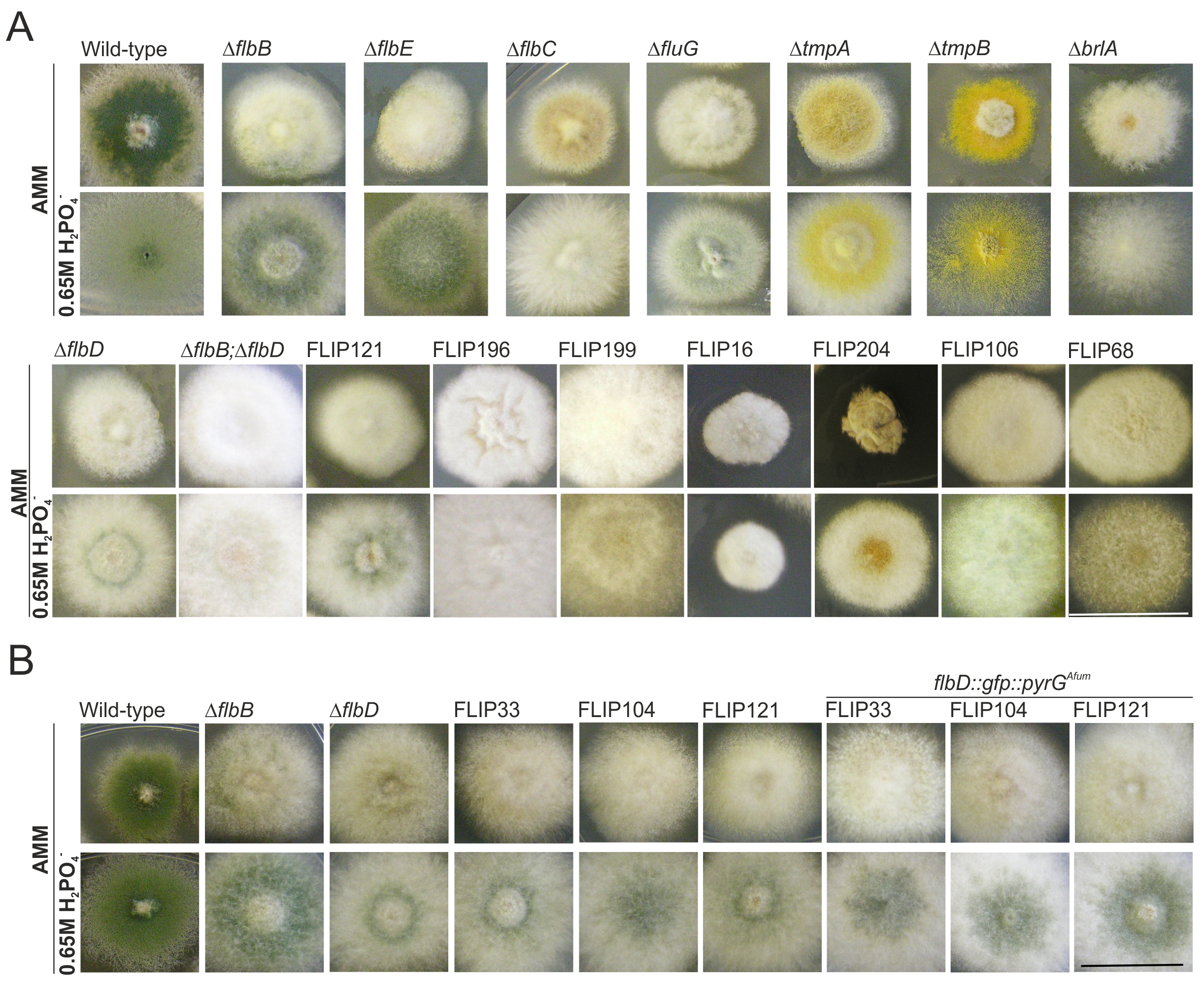

Supplement: Supplementary file 1 [file cells-08-01520-s001.zip › Supplementary_Files/Supp_Figure1_2019_09_27.jpg]

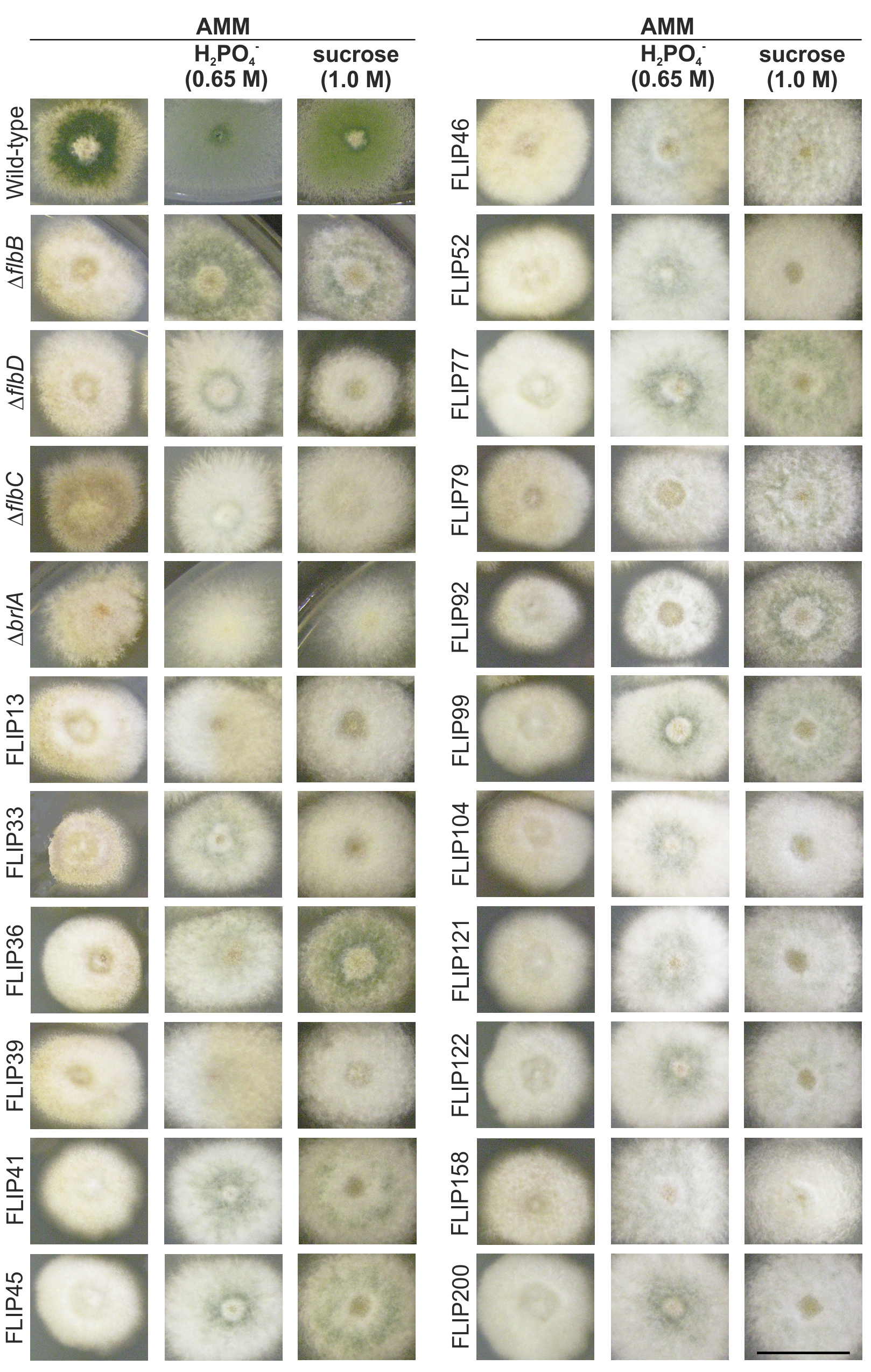

Supplement: Supplementary file 1 [file cells-08-01520-s001.zip › Supplementary_Files/Supp_Figure2_2019_09_27.jpg]

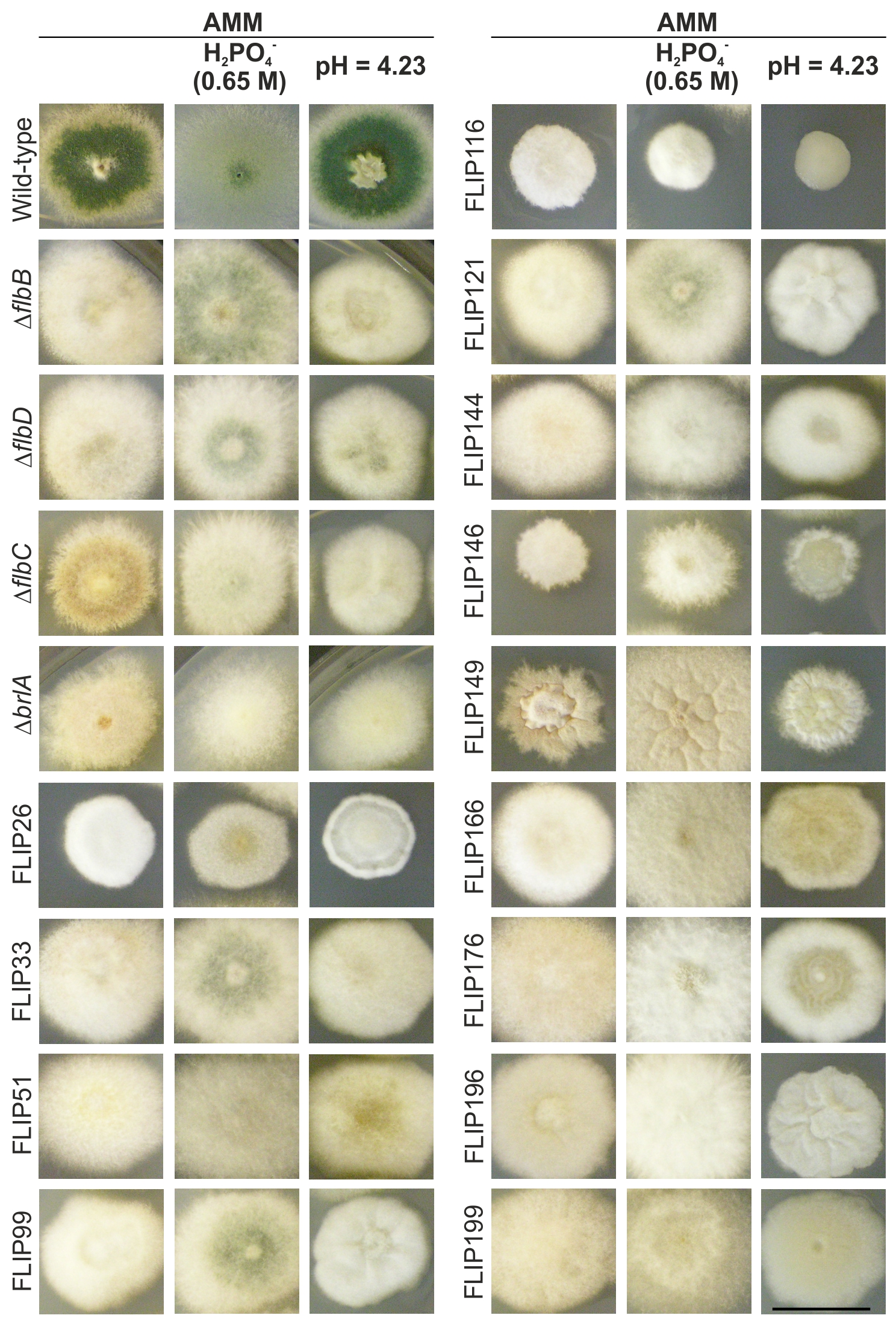

Supplement: Supplementary file 1 [file cells-08-01520-s001.zip › Supplementary_Files/Supp_Figure3_2019_09_27.jpg]

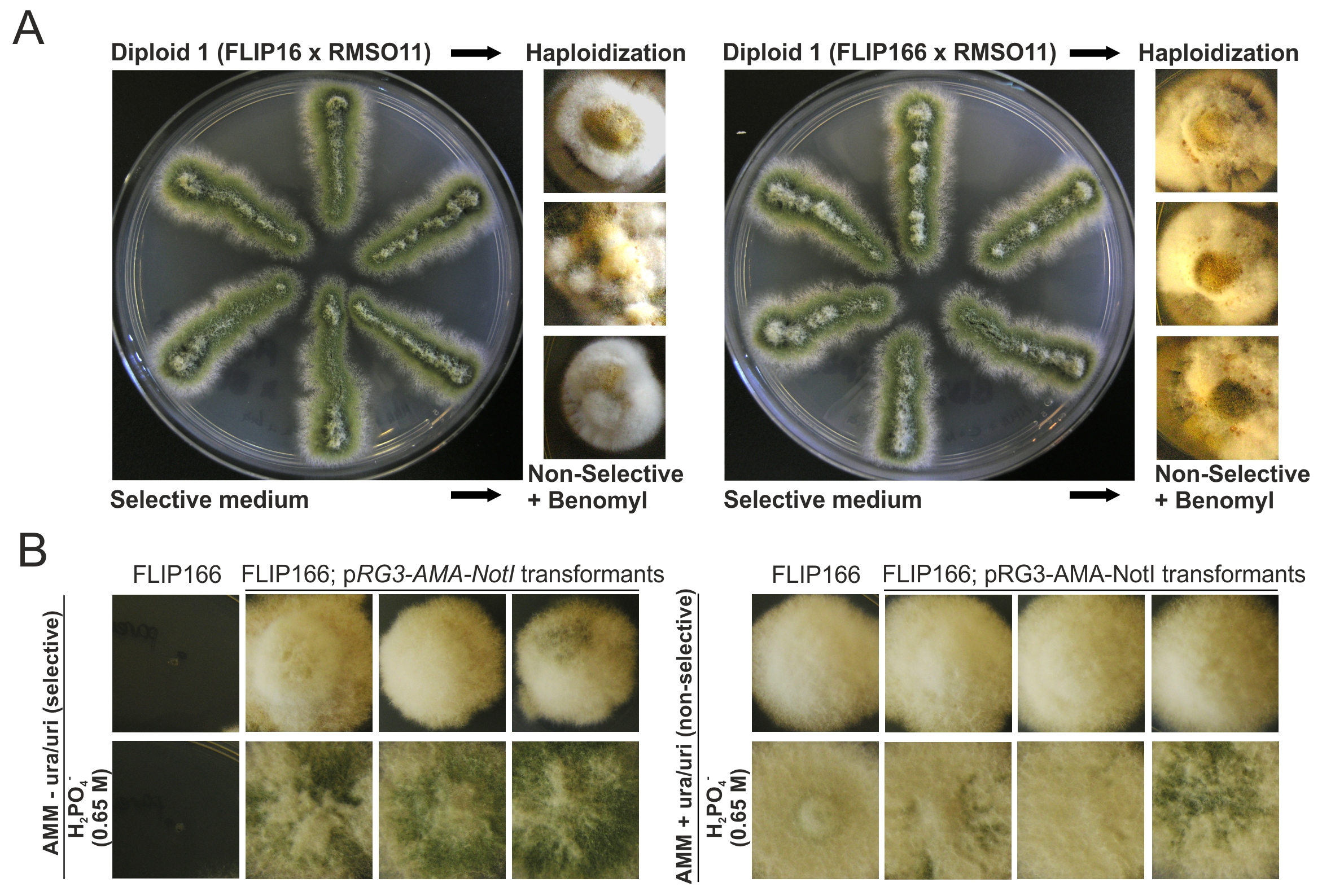

Supplement: Supplementary file 1 [file cells-08-01520-s001.zip › Supplementary_Files/Supp_Figure4_2019_09_27.jpg]
